# Loss-induced Enhanced Transmission in Anisotropic Density-near-zero Acoustic Metamaterials

**DOI:** 10.1038/srep37918

**Published:** 2016-11-25

**Authors:** Chen Shen, Yun Jing

**Affiliations:** 1 Department of Mechanical and Aerospace Engineering, North Carolina State University, Raleigh, North Carolina 27695, USA

## Abstract

Anisotropic density-near-zero (ADNZ) acoustic metamaterials are investigated theoretically and numerically in this paper and are shown to exhibit extraordinary transmission enhancement when material loss is induced. The enhanced transmission is due to the enhanced propagating and evanescent wave modes inside the ADNZ medium thanks to the interplay of near-zero density, material loss, and high wave impedance matching in the propagation direction. The equi-frequency contour (EFC) is used to reveal whether the propagating wave mode is allowed in ADNZ metamaterials. Numerical simulations based on plate-type acoustic metamaterials with different material losses were performed to demonstrate collimation and subwavelength imaging enabled by the induced loss in ADNZ media. This work provides a different way for manipulating acoustic waves.

In recent years, acoustic metamaterials with exotic constitutive parameters have received considerable interest due to their unique features in manipulating acoustic waves^1^,^2^,^3^,^4^,^5^,^6^. Numerous novel applications have been realized by acoustic metamaterials, including acoustic cloaking^7^,^8^, hyperlensing^9^, and energy funneling^10^. Recently, following the concept of epsilon-near-zero (ENZ) metamaterials in electromagnetic waves, density-near-zero (DNZ) metamaterials have been proposed in the acoustic regime, showing exciting potential for controlling acoustic waves^11^,^12^,^13^,^14^. For example, subwavelength imaging and extraordinary transmission using DNZ metamaterials have been proposed^15^,^16^. Elastic metamaterial with anisotropic mass density has also been proposed for the control of elastic waves^17^,^18^,^19^. In ENZ metamaterials, material loss has been demonstrated to introduce transparency, omni-directional collimation, and counterintuitively, improved transmission^20^,^21^. However, little work has been done to investigate the effect of material loss in acoustic ADNZ metamaterials, even though great potential of manipulating acoustic waves using ADNZ metamaterials has been suggested^13^,^15^. In real world applications, material loss is inevitable and its effect on ADNZ metamaterials should be examined. In addition, as we will show in this paper, loss induced in acoustic ADNZ metamaterials may lead to applications such as collimation and subwavelength imaging.

Inspired by the study on ENZ metamaterials, this article will examine the effect of material loss in ADNZ metamaterials where only one component of the mass density tensor is close to zero. The enhanced transmission and collimation effect of ADNZ acoustic metamaterial induced by material loss will be demonstrated. This article shows that when acoustic waves reach an ADNZ metamaterial slab from a certain incident angle, they will bend and be collimated towards the normal direction when a certain amount of material loss is present. The underlying mechanism is discussed, which reveals that high impedance matching condition can be realized by material loss. These effects are verified by the EFC analysis and full wave numerical simulations based on real structures exhibiting anisotropic density-near-zero property.

## Results

### Theory

Consider a homogeneous ADNZ medium whose effective density is positive in the x-direction and near-zero in the y-direction, i.e., *ρ*_*x*_ > 0 and *ρ*_*y*_ → 0^22^. The general dispersion relation for a two-dimensional scenario reads


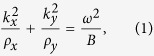


**Figure 1:**
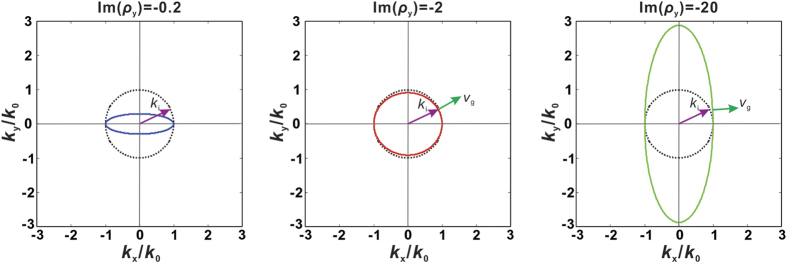
EFC of an ADNZ medium with low, moderate and high losses. The dashed line represents the free space. The incident wave vector *k*_*i*_ has a 30° incident angle.

Where *k* and *ω* are the wave number and angular frequency, respectively, *B* is the scalar bulk modulus. To include material loss in the ADNZ medium, *ρ*_*y*_ is considered a complex number having the form *ρ*_*y*_ = Re(*ρ*_*y*_) + *i*Im(*ρ*_*y*_). Without losing generality, the background medium is assumed to be air with density *ρ*_0_, and *ρ*_*y*_ = *ρ*_*0*_ = 1.2 kg/m^3^. The EFCs of three different cases are plotted in Fig. 1 based on Eq. (1), with the same 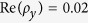 and different losses 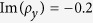, 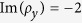, and 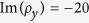. The sign of the imaginary part of the density depends on whether 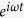 or 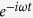 is used. The principle is that the sign should be chosen so that wave decays. These three values correspond to low loss, moderate loss, and high loss, respectively. The EFC of the ADNZ medium is represented by a solid curve and the EFC of free space is represented by a dashed curve for comparison. It can be seen from Fig. 1 that the general EFC of the lossy media is an ellipsoid. When the material loss increases, the major axis of the ellipsoid changes from the horizontal axis to the vertical axis. For an incoming plane wave with incident wave vector *k*_*i*_, incident and transmitted waves should have the identical y-component wave vector due to the conservation of momentum^23^,^24^. When the incident angle is close to zero, the transmitted wave will not be much influenced, as the EFCs of free space and the ADNZ medium touch each other on x-axis. For a larger incident angle, e.g., 30°, it is indicated by Fig. 1 that, for the low loss case, the corresponding *k*_*x*_ inside ADNZ medium has no solutions since no *k*_*y*_ component can be found on the EFC matching the free space *k*_0*y*_ component. However, for the moderate and high loss cases, *k*_*x*_ exists, indicating wave propagation is allowed in the medium. As the group velocity *v*_*g*_ must lie normal to the EFC, for the high loss case, the transmitted wave vector is pointing in the x-direction since the EFC is almost flat. Therefore, the transmitted energy is collimated in an omnidirectional manner towards the normal direction if a large material loss is introduced, which is similar to the ENZ medium with large losses^20^.

**Figure 2:**
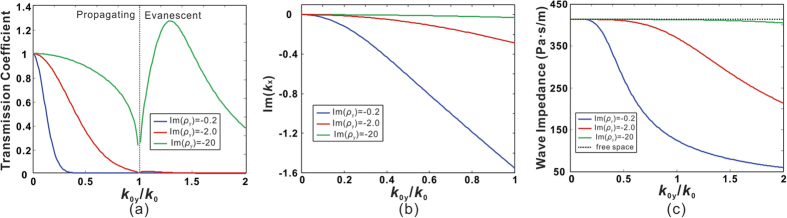
(**a**) Transmission coefficients of the ADNZ medium with various losses. (**b**) The x-direction wave impedance of the ADNZ medium with various losses.

We further investigate the transmission characteristics of the ADNZ slab with various losses. For both propagating and evanescent waves transmitting through an anisotropic layer, the transmission coefficient is given as^15^:


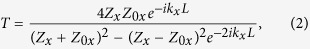


where 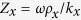 and 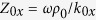 are the wave impedances, *L* is the thickness of the ADNZ slab (that is, along the x-direction), 
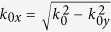
 and *k*_*x*_ are x-component wave vectors in the free space and anisotropic medium, respectively. In the y-direction, the slab is assumed to be infinitely long and 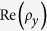 is assumed to be 0.02. The imaginary part of *ρ*_*y*_, which reflects different losses from a homogenized medium perspective, will be included in the effective density to calculate the transmission coefficients from Eq. (2). Figure 2(a) shows the transmission coefficients of both propagating and evanescent waves at 2545 Hz for *L* = 30 cm with effective densities abovementioned used. It can be seen that for normal incidence (*k*_0*y*_ = 0), the transmission coefficients are high in all three cases. This is because the impedance in the x-direction *Z*_*x*_ matches with the free space impedance *Z*_0_, as *k*_0*y*_ is zero. For oblique incidences, especially for large incident angles (large *k*_0*y*_ values), the transmission coefficients for both propagating and evanescent waves increase significantly when more loss is induced. For the low loss case, the transmission coefficient quickly drops to very small values as *k*_0*y*_ increases, implying no propagating mode is allowed in the medium. Note that as illustrated in Fig. 1, sound energy mainly propagates in the x-direction for a large material loss. We hereby evaluate the values of 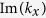 and *Z*_*x*_ in terms of 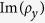. Since at the interface of the ADNZ medium and free space, we have 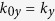 due to conservation of momentum^24^, the wave vector *k*_*x*_ can be obtained by:


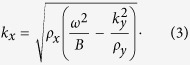


The wave impedance *Z*_*x*_ is thus:


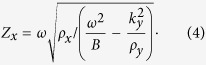


By inserting complex *ρ*_*y*_ into Eq. (4), the dependence of 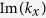 and *Z*_*x*_ on 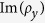 is shown in Fig. 2(b,c). As a reference, the free space impedance 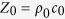 is also included in the Fig. 2(c). It can be clearly observed that for large material losses, 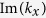 becomes smaller, indicating less attenuation in the x-direction. The impedance matching condition is also fulfilled within a broad range of *k*_0*y*_ starting from 0. These results are not surprising, as we can observe higher transmission in Fig. 2(a) for a certain *k*_0*y*_ value with large losses. We also note that the enhanced transmission when *k*_0*y*_ > 1 implies that the evanescent components can be coupled through an ADNZ slab and is favorable for subwavelength imaging applications^25^.

### Numerical simulations

**Figure 3:**
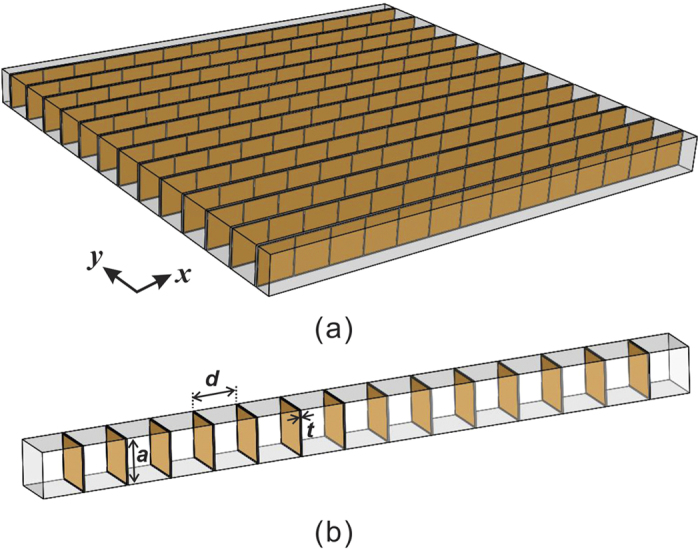
(**a**) 2D ADNZ metamaterial (**b**) y-direction of the ADNZ medium, where the real part of the effective density is close to zero around the first resonance frequency of the plate. The imaginary part is tuned by the material loss.

To verify the proposed phenomenon with numerical simulations, we construct the ADNZ medium utilizing plate-type acoustic metamaterials, which yield negative density below a cut-off frequency and a near-zero-density around the cut-off frequency (i.e., the first resonance frequency of the plate). It should be noted that, other candidates which could generate near-zero-density (e.g., space-coiling metamaterials) will have similar effects as long as a certain amount of loss is introduced. The two-dimensional (2D) ADNZ metamaterial is depicted in Fig. 3(a), where periodically arranged, clamped square plates facing the y-direction are placed inside a 2D waveguide. The plate is assumed to be made of paper and has the same material property as that in an earlier study^22^. Although the same structure is used here, the losses in the thin plates were ignored in^22^. Their effects, however, can be significant in certain cases as will be demonstrated in this study. The theory presented here can also be generalized to other unit cell candidates, such as labyrinthine type metamaterials^2^. Different material losses are now considered to give rise to complex effective density in the y-direction. The Poisson’s ratio, density, width and thickness for the plate are 0.33, 591 kg/m^3^, 20 mm and 0.3 mm, respectively. Since wave propagation inside this structure can be decoupled in the x- and y-directions^3^, the effective properties in these two directions can be estimated separately. To this end, one-dimensional (1D) models are first utilized to study the effective density of the ADNZ metamaterial. Numerical simulations based on finite element analysis are carried out to verify our theories. Since there are no plates in the x-direction, the effective density along the x-direction is considered as that of air^22^, and no loss is considered, i.e., *ρ*_*x*_ = 1.2 kg/m^3^. The bulk modulus is also assumed to be the same as air since it will not be altered by the thin plates^26^,^27^. The setup for evaluating the complex effective density of the ADNZ metamaterial in the y-direction is shown in Fig. 3(b), where periodically arranged plates are placed in a square waveguide with width *a* = 20 mm. Each plate is separated by a distance *d* = 20 mm. To include material loss in the simulations, the Young’s moduli of the plates are set to be complex numbers, i.e., 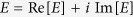. The same real parts of the Young’s moduli are used, i.e., 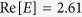
GPa. Three values are chosen for the imaginary parts: 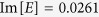
GPa, 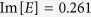
GPa and 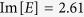
GPa. The corresponding loss factors (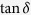) are 0.01, 0.1, and 1. They correspond to low, moderate and high loss case, respectively. As can be seen below, the complex Young’s modulus will translate to the complex effective density via the homogenization process.

**Figure 4:**
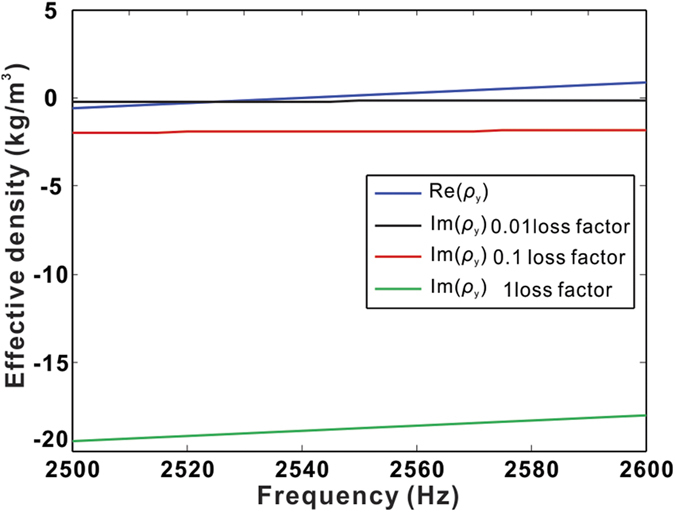
Effective density along the y-direction with different losses.

A lumped model is used to estimate the effective density in the y-direction, with expression 
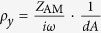
22, where 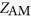 is the acoustic impedance of the plate, 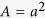 is the cross-sectional area of the waveguide. The acoustic impedance of the square plate used in the simulation is calculated as 
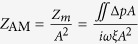
. The pressure difference across the plate Δ*p*, and the average transverse displacement of the plate *ξ* are estimated numerically in order to get the mechanical impedance of the plate *Z*_*m*_. Figure 4 depicts the calculated effective density of the three cases. Since the real part is loss-independent, it is only represented by a single curve^28^. It can be found from Fig. 4 that with higher material loss, the absolute value of the imaginary part of *ρ*_*y*_ increases. The real part of *ρ*_*y*_ is near zero (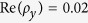) around 2545 Hz and the corresponding 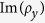 is −0.2, −1.9, and −18.8, respectively.

**Figure 5:**
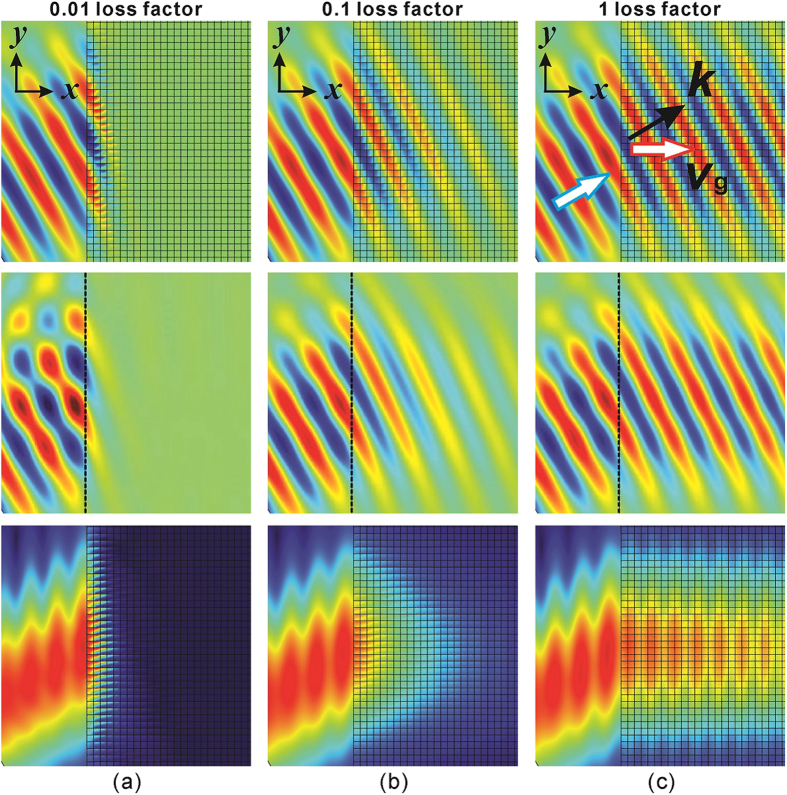
The acoustic pressure and intensity fields. Top: acoustic pressure fields using real structure. Middle: acoustic pressure fields using effective medium, dotted lines denote the interface of free space and ADNZ medium. Bottom: acoustic intensity fields. (**a**) Low loss. (**b**) Moderate loss. (**c**) High loss. Collimation and enhanced transmission are clearly observable in the high loss case.

Full wave simulations based on effective medium and real structures are carried out to study the enhanced transmission for loss-induced ADNZ metamaterials. A Gaussian beam with frequency 2545 Hz is transmitted with an incident angle 30° to the ADNZ metamaterial slab. The corresponding acoustic pressure and intensity fields are plotted in Fig. 5. It is clear that for the low loss case, the incident wave cannot excite propagating modes inside the ADNZ medium and the acoustic energy vanishes quickly inside the slab. When the loss increases, higher transmission is observed, and the acoustic energy is collimated in the x-direction, which is well predicted by the theory presented above. This seems to be counterintuitive, as more material losses increase the transmission. However, a rigorous analysis of the EFC shows that in the high loss case, the acoustic waves are forced to travel along the x-direction (Fig. 1(c)), where there are no plates and therefore no energy loss in that direction (Fig. 5(c)). This is consistent with the fact that the homogenized acoustic medium does not yield loss in the x-direction.

**Figure 6:**
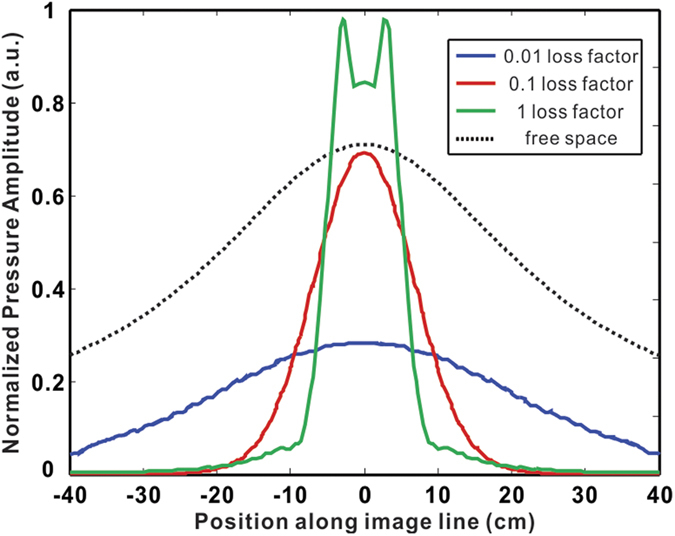
Pressure amplitude distribution on the image plane for different loss factors.

Finally, to demonstrate acoustic imaging with resolution below the diffraction limit, two square acoustic sources with width 2 cm are placed in front of the ADNZ slab with thickness 30 cm, corresponding to 2.23 λ (λ is the wavelength) at 2545 Hz. The separation of the sources is 6 cm, corresponding to 1/2.25 λ. The resulting normalized acoustic pressure amplitude distribution on the other side of the ADNZ slab is depicted in Fig. 6 with different loss factors. Two peaks can be clearly resolved for the high loss case, owing to the enhanced transmission of evanescent waves. The pressure amplitude profile shows only one peak for other cases, as the evanescent wave energy cannot be coupled through the ADNZ slab, as indicated by Fig. 2. It should be stressed that the structure used here for the ADNZ slab only serves as an example to achieve subwavelength imaging. To improve its performance, smaller unit cells can be used for better homogenization and the thickness of the ADNZ slab can also be reduced for better imaging quality.

## Discussion

While the material losses in passive acoustic metamaterials are largely ignored in most studies, its effect on wave confinement and enhanced transmission are demonstrated in this article, which could lead to useful applications such as subwavelength imaging. The enhanced transmission phenomenon in loss-induced ADNZ acoustic metamaterials is studied based on effective medium and plate-type acoustic metamaterials. Theoretical analysis revealed that the enhanced transmission is due to the enhanced propagating and evanescent wave modes and better matched wave impedance. The collimation effect can be realized by introducing a large material loss and can be further utilized for subwavelength imaging. Numerical simulations were conducted to verify the theory using plate-type acoustic metamaterials and the material loss is included by introducing complex Young’s modulus. Although similar effects may occur by introducing a large density contrast in orthogonal directions in the 2D anisotropic metamaterial (e.g., increase the real part of the effective density), our approach here does not change the real part of the ADNZ medium and hence the intriguing phenomena associated with near-zero-density can be preserved. Moreover, the material loss can serve as an alternative means of tuning the effective parameters of the acoustic metamaterials as the real part of the effective densities may not always be convenient to be adjusted. For instance, to achieve subwavelength imaging, the modulation of both real and imaginary part of the medium can be combined for better performance. For other applications utilizing ADNZ medium, our paper shows that the loss-free treatment may not be correct, since the effect of loss such as bending may stand out when some material loss is considered. The findings in this paper may find applications in directional sensing and acoustic imaging.

## Method

The numerical simulations in this article are performed by using commercial finite element package COMSOL Multiphysics. The Acoustic-Structure Interaction module is utilized for simulations of structures containing clamped plates. The density and speed of sound in the background medium (air) are 1.2 kg/m^3^ and 343 m/s, respectively. Perfect matched layers (PMLs) are used to minimize the field distortion caused by the reflections on the boundaries.

## Additional Information

**How to cite this article**: Shen, C. and Jing, Y. Loss-induced Enhanced Transmission in Anisotropic Density-near-zero Acoustic Metamaterials. *Sci. Rep.* **6**, 37918; doi: 10.1038/srep37918 (2016).

**Publisher’s note:** Springer Nature remains neutral with regard to jurisdictional claims in published maps and institutional affiliations.
